# Transient Thermal Response and Simulation-Driven Machine Learning Prediction for Temperature in a Petroleum Coke Vertical Shaft Calciner

**DOI:** 10.3390/ma19214569

**Published:** 2026-10-29

**Authors:** Longxin Lai, Jinlong Zhong, Jing Li, Jindi Huang

**Affiliations:** 1 School of Metallurgical Engineering, Jiangxi University of Science and Technology, Ganzhou 341000, China; 6120250206@mail.jxust.edu.cn (L.L.); z835720916jl@163.com (J.Z.); 9120200008@jxust.edu.cn (J.L.); 2 Jiangxi Provincial Key Laboratory of High-Performance Steel and Iron Alloy Materials, Jiangxi University of Science and Technology, Ganzhou 341000, China; 3 Jiangxi Provincial Center for Interdisciplinary Studies in Future-oriented Materials, Jiangxi University of Science and Technology, Ganzhou 341000, China

**Keywords:** simulation, vertical shaft calciner, petroleum coke, machine learning, transient temperature

## Abstract

Transient temperature evolution in a petroleum coke vertical shaft calciner (VSC) helps assess responses to operating disturbances, but transient multiphysics simulations are costly. A transient two-dimensional multiphysics model representing one pot and the flues on one side of a 24-pot, eight-layer co-current VSC, with prescribed flue temperatures, was developed to investigate step changes in flue temperature and per-pot discharge rate. Raising the upper-flue temperature by 200 K extended the high-temperature calcination zone from 1.3 to 1.9 m and shifted it upward. Increasing the discharge rate from 95 to 115 kg/h shortened this zone from 1.3 to 0.3 m and shifted it downward. The furnace wall approached a quasi-steady state within 10 h, whereas the coke bed required up to 40 h. Using 4.59 × 10^5^ numerically generated temperature values from 510 pot-centerline locations under nine flue-temperature disturbance conditions, a Temporal Convolutional Network–Bidirectional Long Short-Term Memory–Convolutional Neural Network–Attention (TCLCA) surrogate model was developed. TCLCA achieved an *R*^2^ of 0.997 in recursive prediction while reducing computational time by approximately 94%. The framework enables rapid prediction of simulated centerline temperature evolution.

## 1. Introduction

The VSC is a key piece of equipment for calcining petroleum coke in China. The primary application of the resultant calcined coke is in the fabrication of prebaked carbon anodes used in aluminum electrolysis [1,2]. The calcination process itself involves subjecting raw petroleum coke to elevated temperatures to eliminate volatile components [3,4,5]. This thermal treatment enhances the material’s structural density and removes undesirable impurities, thereby yielding a product that satisfies the critical specifications for electrolytic anode production.

The operating temperature inside the furnace is a decisive parameter. It significantly influences process efficiency, energy consumption, and final product quality [6,7]. Precise thermal management is, therefore, directly linked to key production metrics. Presently, conventional VSC calcination operations encounter several technical shortcomings. These challenges encompass suboptimal strategies for recirculating calcined coke, inadequate harnessing of volatile matter released during heating, low efficiency in recovering waste heat, and abrupt disturbances in the internal temperature distribution caused by sudden operational shifts [3,8].

A fundamental challenge is the difficulty of capturing real-time, transient changes in the furnace’s temperature field. This limitation frequently results in operational adjustments that are both delayed and imprecise. The consequences manifest as increased carbonaceous material loss, accelerated wear of refractory linings, and diminished quality consistency of the calcined output [9,10]. Current mitigation approaches often depend on manual intervention guided by operator expertise to tune process variables, such as fuel gas flow rate and composition, solid discharge rate, draft pressure in the flues, and feedstock particle size [11,12,13,14].

However, comprehensive monitoring of temperature variations across the entire furnace volume in real time remains impractical, constrained by both technical sensor limitations and economic considerations [15,16]. While computational fluid dynamics (CFD) simulation provides a powerful tool for accurate prediction of intricate heat and mass transfer phenomena and facilitates analysis of multi-parameter coupling effects, its application is hampered by substantial computational expenses and time requirements. Consequently, CFD alone cannot deliver the immediate feedback necessary for real-time production control.

CFD provides a useful approach for investigating the thermal behavior of vertical shaft calciners (VSCs), although transient simulations can be computationally expensive. Xiao et al. [2] developed a real-time two-dimensional mathematical model for predicting temperature distributions in petroleum coke VSCs. Zhou et al. [17] investigated the flow and temperature distributions in a petroleum coke calciner using a coupled fluid–solid CFD model and compared the calculated pressure distribution with plant measurements. Meanwhile Xiao et al. [18] analyzed petroleum coke heating using a two-fluid model. Li et al. [19] examined the effects of operating parameters on the migration of the high-temperature calcination zone, and Zheng [20] developed a three-dimensional unsteady two-fluid model to examine the effects of volatile inlet configurations, excess air coefficients, and flue outlet pressure on furnace thermal behavior, with validation against measured flue temperatures.

To improve computational efficiency, simulation-driven machine-learning methods have been applied to temperature prediction in rotary kilns [21], internal-state prediction in blast furnaces [22], and temperature uniformity optimization in wafer annealing furnaces [23]. These studies demonstrate the potential of combining numerical simulations with machine learning for efficient prediction in industrial thermal processes. A comparison of the relevant studies and their research scopes is presented in Table 1.

As summarized in Table 1, previous studies have investigated furnace thermal behavior, calcination-zone migration, and computationally efficient temperature prediction. However, further investigation is needed to characterize the time-dependent migration of the high-temperature calcination zone and the differences in thermal response times between the furnace wall and coke bed following step changes in operating parameters. In addition, the integration of multiphysics-based transient analysis with machine-learning-based recursive prediction of centerline temperature evolution in petroleum coke VSCs remains relatively limited.

To address these research needs, the present study develops a transient two-dimensional multiphysics simulation model representing a pot and its adjacent flues to investigate temperature evolution, calcination-zone migration, and thermal response times under step changes in flue temperature and per-pot discharge rate. Furthermore, TCLCA is developed using numerically generated temperature sequences under different operating conditions to enable rapid recursive prediction of pot-centerline temperatures. Its predictive accuracy and computational efficiency are evaluated against numerical simulation results and alternative machine-learning models.

## 2. Mathematical Model and Numerical Methods

### 2.1. Modelling Assumptions

The calcination of petroleum coke in VSC involves multiple coupled physical processes and chemical reactions. These processes involve petroleum coke descent, heat and mass transfer, and volatile release within the furnace.

To simplify the analysis of transient temperature field distribution, the following assumptions are made for the model:

(1) The simulation domain represents a quarter-sectional view of one pot group. The cooling water jacket region is ignored. Heat loss from the external furnace surface was neglected, whereas heat conduction through the refractory wall was included.
(2) Petroleum coke is represented as a continuous medium for the calculation of heat transfer and volatile transport within the pot.
(3) Petroleum coke descends continuously through the pot at a prescribed velocity determined by the per-pot discharge rate, as defined in Equation (3). The momentum equation is not explicitly solved.
(4) The effect of pyrolytic gas flow on the downward movement of solid petroleum coke is disregarded.
(5) The thermal conditions of the flues are represented by prescribed temperatures for individual flue layers, with a uniform temperature assumed along each corresponding boundary. The prescribed flue temperatures are independent of variations in volatile release.
(6) The progress of the pyrolysis reaction is controlled by chemical reaction kinetics. And the endothermic effects of moisture evaporation and petroleum coke pyrolysis are approximately accounted for using the effective heat capacity method, whereby the associated enthalpy changes are incorporated into the effective specific heat capacity of petroleum coke.

Under these assumptions, the numerical model focuses on transient heat transfer, species transport, and pyrolysis under prescribed coke-velocity and flue-temperature conditions.

### 2.2. Governing Equations

The computational domain of the VSC model is divided into two regions: the pot and the furnace body. The pot region describes the vertical descent of petroleum coke particles and the pyrolysis process of volatile matter through governing equations. The furnace body region simulates the heat transfer characteristics of the heterogeneous solid-phase zone composed of refractory bricks (silica bricks) using the energy conservation equation.

The solid-phase mass conservation equation in the pot region is given by the following:

(1) $$\frac{\partial (\alpha \rho_{s})}{\partial t}+\nabla \cdot (\alpha \rho_{s}u)=-\sum_{i=1}^{m}R_{i}$$

*R_i_* = *αρ*_s_c*_i_r_i_* (2)

where **u** is solid-phase velocity vector, *⍴_s_* is the density of raw petroleum coke, *R_i_* is the mass source term of volatile release during coke pyrolysis, *α* is the solid-phase filling rate, satisfying *α* = 1 − *ε_s_*, where *ε*_*s*_ is the void fraction of the packed petroleum coke, *m* is the total number of volatile components and *r_i_* is the corresponding pyrolysis reaction rate.

The initial velocity at the inlet (*v_s__0_*) is expressed as follows:

(3) $$v_{s0}=\frac{Q_{m}}{\alpha \rho_{s}A_{c}},u_{s0}=0$$

where *v_s0_* is the initial longitudinal (y-direction) velocity of the solid phase, *Q_m_* is the mass flow rate of raw coke, *A_c_* is the cross-sectional area of the pot, and *u_s0_* is the initial transverse (x-direction) velocity of the solid phase. The transient transport of volatile pseudo-components and moisture was described by Equation (4), with molecular diffusion neglected.

(4) $$\alpha \rho \frac{\partial c_{i}}{\partial t}+\alpha \rho u\cdot \nabla c_{i}=S_{i}$$

where *c_i_* is the concentration of component *i*, and *S_i_* is the corresponding reaction source term.

The heat transfer process is governed by the equation:

(5) $$\alpha \rho C_{p}\frac{\partial T}{\partial t}+\alpha \rho C_{p}u\cdot \nabla T-\nabla \cdot \left(K_{\mathrm{eff}}\nabla T\right)=0$$

where *C_p_* is the effective heat capacity, *T* is the absolute temperature, *K*_eff_ is the effective thermal conductivity.

The main materials in this model include petroleum coke and furnace lining materials. The key physical property parameters for petroleum coke and silica bricks are listed in Table 2, where *d_p_* represents the equivalent particle size of petroleum coke particles.

The pyrolysis of petroleum coke was described using the three-stage independent parallel reaction kinetic model reported by Huang et al. [25]. The model consists of three pseudo-components, whose mass fractions and kinetic parameters are presented in Table 3. The pyrolysis reaction rate of each pseudo-component is expressed as follows:

(6) $$r_{i}=A\exp\left(-\frac{E_{i}}{\mathrm{RT}}\right){\left(1-\xi \right)}^{n_{i}}$$

where *r_i_* is the pyrolysis reaction rate of pseudo-component *i*; *A* is the pre-exponential factor; *E_i_* is the activation energy; *R* is the universal gas constant; *ξ* is the pyrolysis conversion degree; and *n_i_* is the reaction order.

The proximate and ultimate analyses of petroleum coke were taken from Huang et al. [25]. The reported composition includes 3.00 wt.% moisture, 10.48 wt.% volatile matter, and 0.25 wt.% ash, with elemental contents of 86.98 wt.% C, 2.81 wt.% H, 1.28 wt.% O, 1.39 wt.% N, and 0.32 wt.% S. The relative mass fractions and reaction-rate expressions of the three volatile pseudo-components are presented in Table 3.

The model describes the overall release of volatile matter through three pseudo-components, with moisture release considered separately. The chemical compositions of individual gaseous products are not explicitly resolved.

### 2.3. Initial and Boundary Conditions

The transient simulations were initialized using the stationary solution obtained under the baseline operating conditions. Accordingly, the initial temperature and species concentration fields were prescribed as follows:

*T*(*x*,*y*,0) = *T*_ss_(*x*,*y*),*c_i_*(*x*,*y*,0) = *c_i_*_,ss_(*x*,*y*) (7)

where the subscript ss denotes the steady-state solution obtained under the baseline operating conditions.

The initial and boundary conditions are summarized in Table 4. Prescribed temperatures were applied to the eight flue layers (L1–L8), while the remaining thermal and species-transport boundary conditions were assigned to their corresponding boundaries.

### 2.4. Numerical Solution Methods

The governing equations were solved using COMSOL Multiphysics 6.4 based on the finite element method. A stationary calculation was first performed to obtain the baseline solution, which was subsequently used to initialize the transient simulations. Both the heat-transfer and species-transport equations were included in the time-dependent calculation. The simulation time was set to 0–5000 min, with results output at 1 min intervals. The computational mesh and mesh independence study are presented in Section 3.2.

The temporal discretization employed an implicit backward differentiation formula with adaptive time stepping and variable orders of 1–2. The relative tolerance was set to 5 × 10^−3^, and the absolute tolerances were controlled using a scaling factor of 0.1. The nonlinear equations were solved using a fully coupled Newton method with a damping factor of 0.9 and a maximum of eight iterations. A solution-based termination criterion was adopted, and the PARDISO direct solver was used for the linear systems.

The spatial discretization and stabilization settings were specified according to the finite-element formulations implemented in the heat-transfer and species-transport interfaces. No pressure–velocity coupling algorithm was required because the coke velocity was prescribed rather than obtained from an independently solved momentum equation.

## 3. Model Parameters

### 3.1. Geometric Structure

**Figure 1:**
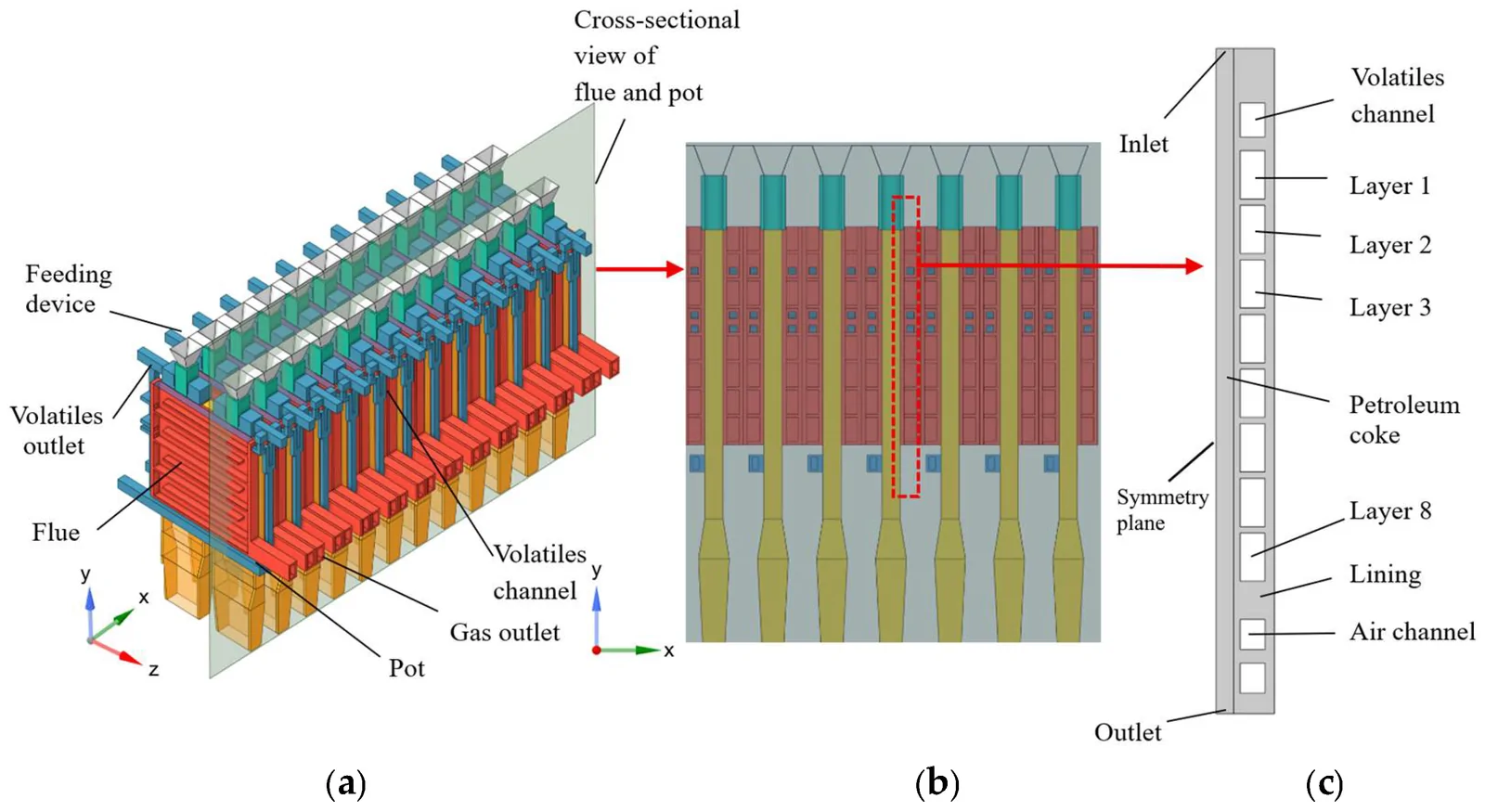
Structural diagram of the vertical shaft calciner (VSC): (**a**) 3D structural schematic diagram; (**b**) 2D structural schematic diagram of the VSC; (**c**) 2D simulation model of a representative pot and adjacent flues. The blue, red, yellow, and green regions represent the volatile channels, flues, pots, and feed inlets, respectively.

The three-dimensional structure of the VSC is shown in Figure 1a. Its main structure is arranged symmetrically in double rows along the XY plane. Each group of pots operates in parallel, and adjacent pots are connected via a shared flue system to ensure uniform heat distribution.

To investigate how changes in different operating parameters affect the transient temperature field distribution during the calcination process in the VSC, the complex furnace structure was reasonably simplified. A single pot was selected as the research object. A sectional view, extending from the inlet to the outlet and including the flues on one side, was extracted to construct the two-dimensional model shown in Figure 1c. The flue region includes a top volatile channel, a bottom air channel, and eight layers of flues, all designed as hollow structures. To simplify calculations, the flue temperatures are directly set as known values. Raw petroleum coke undergoes feeding, heating, and calcination processes, finally settling into the discharge outlet after calcination is complete.

During modeling, to highlight the core heat transfer characteristics, the influence of inspection holes and soot blowing holes on the flue gas region was neglected. The water jacket located below the pot, which has a relatively weak impact on heat transfer between the pot and the flues, was omitted to reduce computational complexity. The specific geometric parameters of the pot and flues are detailed in Table 5.

### 3.2. Mesh Generation, Independence Verification and Model Validation

**Figure 2:**
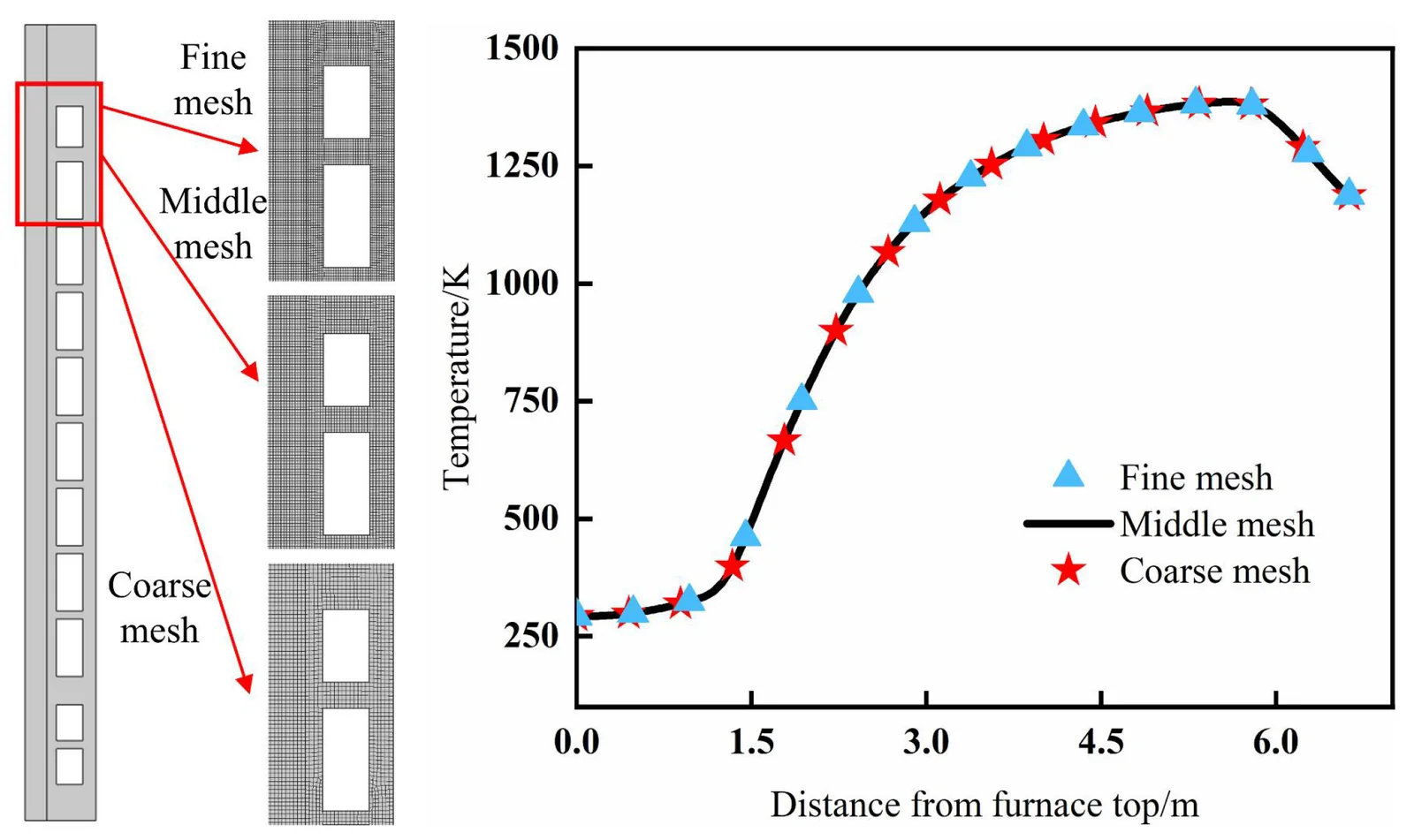
Mesh schematic and temperature curve distribution of mesh center.

Mesh size is a critical parameter affecting simulation accuracy and computational time. To verify the sufficiency of the mesh, simulations were conducted using three different mesh densities: a fine mesh (21,310 cells), a medium mesh (16,380 cells), and a coarse mesh (8305 cells). The temperature profiles along the central axis of the pot for these three meshes are shown in Figure 2. The results from the coarse, medium, and fine meshes are virtually identical, with an average error of less than 0.1%.

To ensure high computational accuracy, efficiency, and to obtain a sufficient dataset of the temperature field in both the pot and flue regions, the medium mesh was selected for all subsequent simulations. The mesh consists of predominantly quad-dominant cells.

### 3.3. Baseline Thermal Characteristics

**Figure 3:**
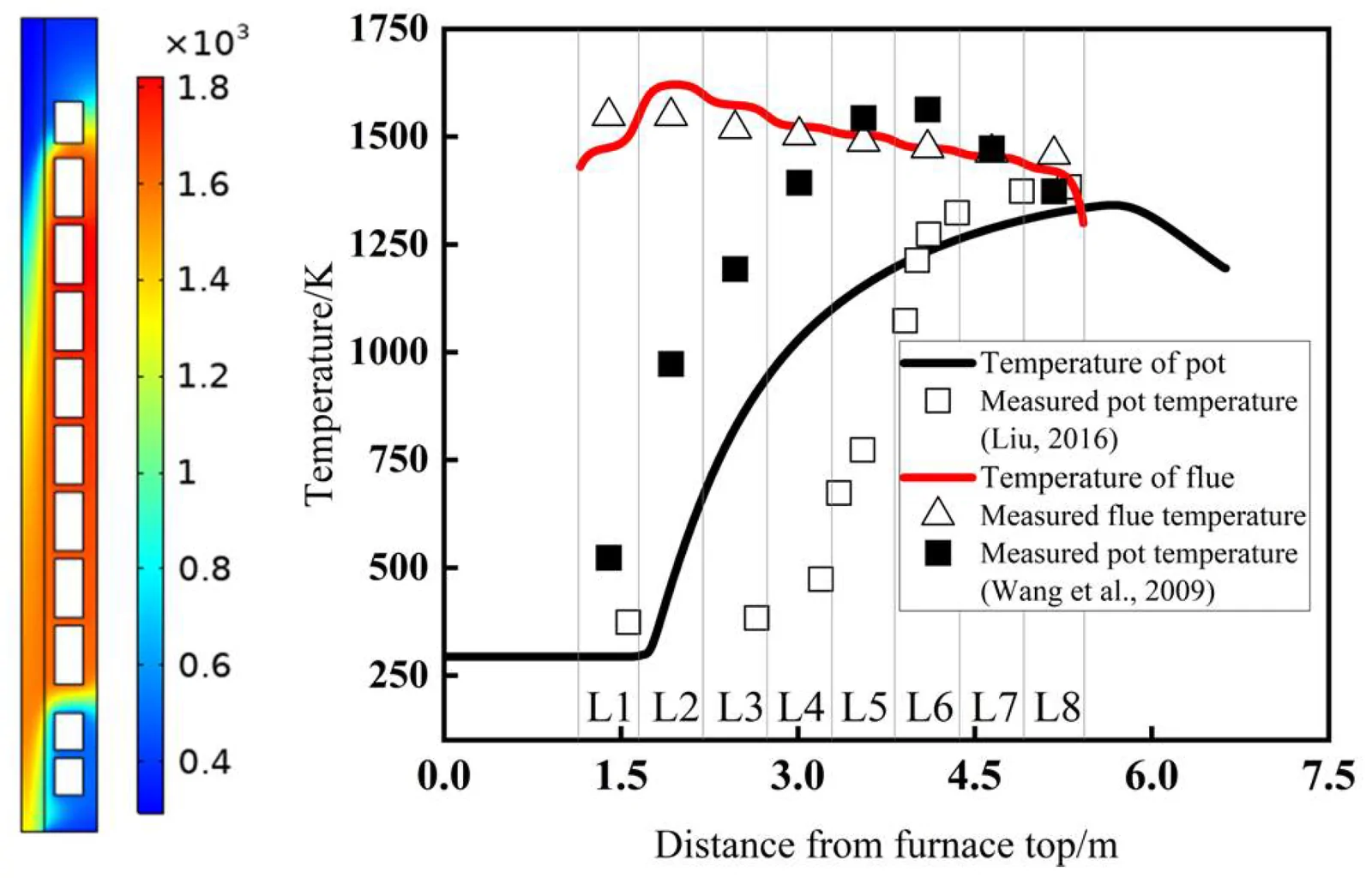
Temperature contour and axial simulation temperature profiles of the pot and flue versus vertical distance from furnace top. The measured pot temperature data are from Liu [26] and Wang et al. [7].

Figure 3 compares the simulated axial temperature distributions under baseline operating conditions with the temperature measurements reported in Refs. [7,26]. The prescribed flue-temperature profile is generally consistent with the reported measurements, supporting the selection of the thermal boundary conditions. However, noticeable discrepancies in petroleum coke temperature occur in the L3–L5 regions. These deviations may be partly attributed to the plug-flow assumption, which neglects local variations in coke descent velocity and residence time, and the two-dimensional approximation, which does not explicitly resolve three-dimensional thermal variations. In addition, uncertainties in the actual measurement locations may result in differences between the reported temperatures and the simulated pot-centerline temperatures. Notably, the predicted centerline temperature profile generally lies between the two sets of reported coke-temperature measurements over much of the L3–L5 region. This comparison provides qualitative support for the predicted axial heating trend, although the remaining discrepancies indicate limitations in the local predictive accuracy of the simplified numerical model.

**Figure 4:**
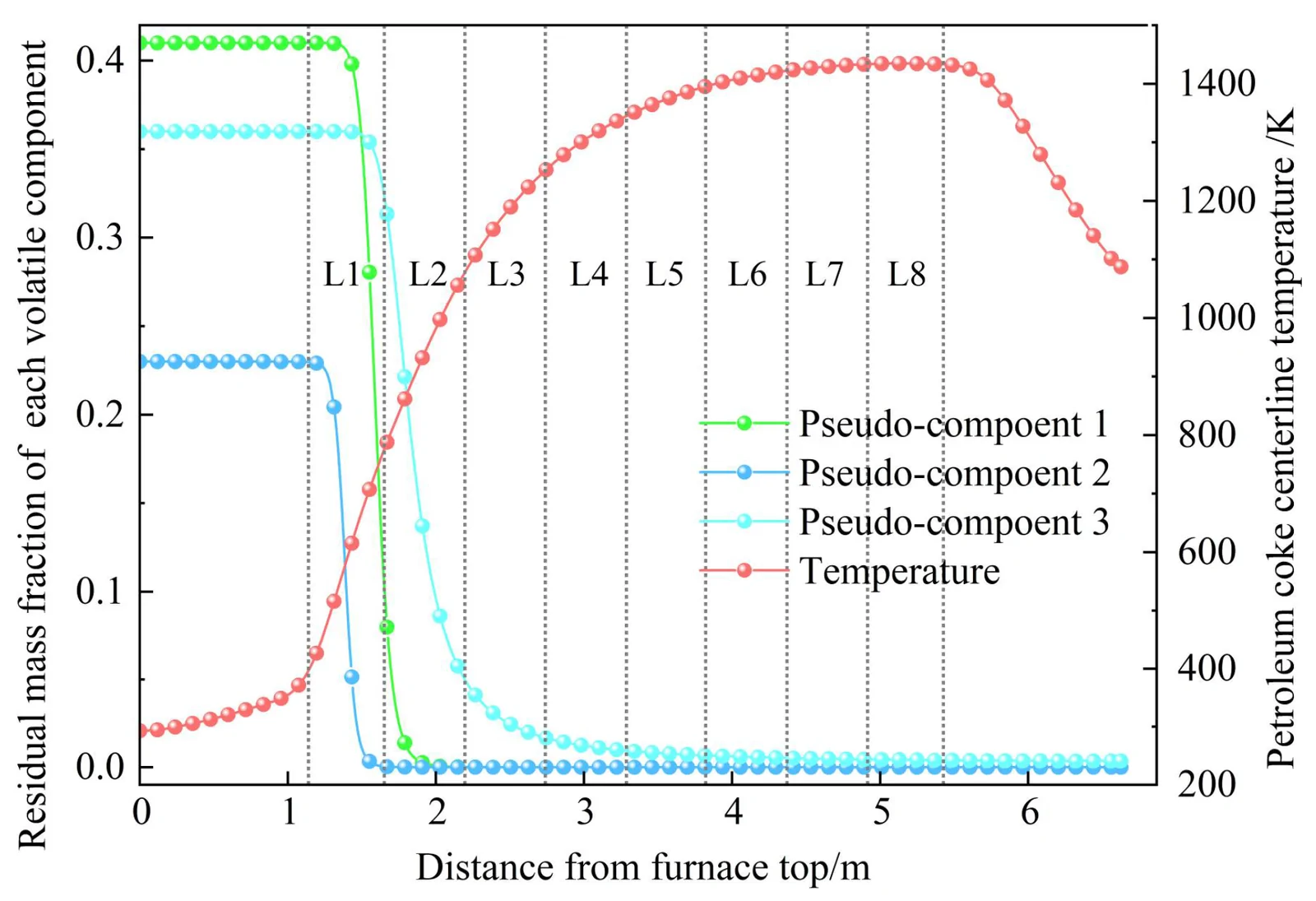
Axial distributions of the residual contents of the three volatile pseudo-components and petroleum coke temperature along the pot centerline.

Figure 4 presents the axial distributions of the residual contents of three volatile pseudo-components and the petroleum coke temperature along the pot centerline. As shown in Figure 3 and Figure 4, the large temperature difference between the incoming petroleum coke and the adjacent L1 flue leads to rapid heating as the coke descends. The flue temperature reaches its maximum in the L2 region, providing a strong thermal driving force for coke heating and volatile release. Under the prescribed flue-temperature conditions, the increasing coke temperature promotes the progressive release of the three pseudo-components.

The release characteristics differ among the pseudo-components. Pseudo-component 2 decreases rapidly between approximately 1.2 and 1.6 m below the furnace top, corresponding to coke temperatures of approximately 400–800 K. Pseudo-component 1 is mainly released between approximately 1.4 and 1.8 m, over a temperature range of approximately 600–1000 K. In contrast, pseudo-component 3 exhibits a broader release region, with its residual content decreasing sharply between approximately 1.6 and 2.5 m as the coke temperature rises from approximately 800 to 1200 K. Most of pseudo-component 3 is released by approximately 2.5–3.0 m, although a small residual fraction persists farther down the pot. As the coke descends through the L3–L6 regions, the decreasing temperature difference between the flues and the pot results in a slower heating rate. In the lower L7–L8 regions, the coke reaches the high-temperature calcination range, while the residual contents of the volatile pseudo-components remain close to zero.

## 4. Discussion of Simulation Results

### 4.1. Impact of Sudden Flue Temperature Change

**Figure 5:**
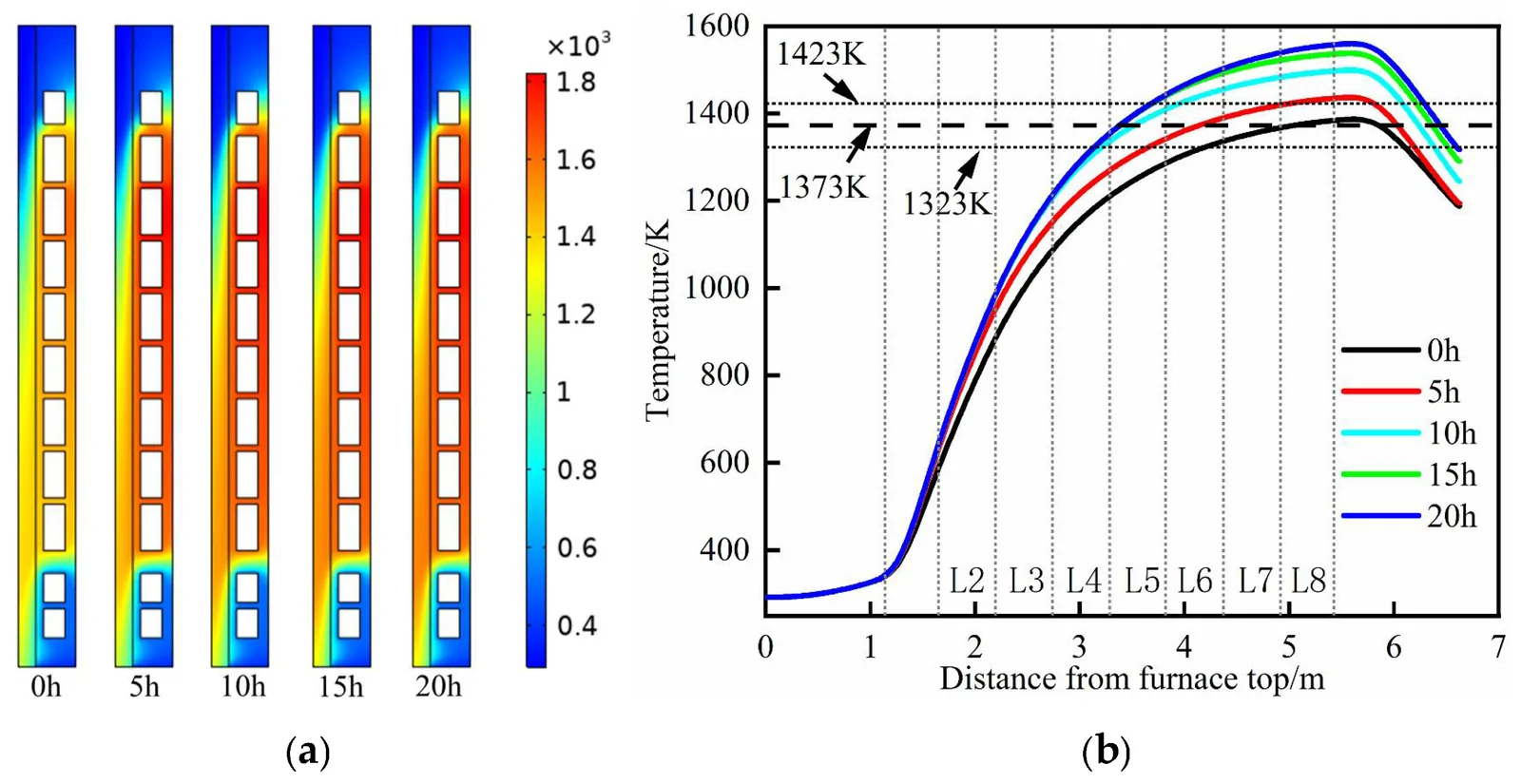
The temperature distribution of the model and the central temperature curve of the pot after the temperature of the flue rises by 200 K: (**a**) model temperature distribution, (**b**) central temperature curve of pot.

Figure 5 shows the temperature contour and the temperature curves at the center of the pots after the flue temperature is increased by 200 K from the baseline condition. When the flue temperature increases abruptly by 200 K, the flue wall temperature rises rapidly within 5 h due to its high thermal conductivity and stabilizes after approximately 10 h. Concurrently, the high-temperature calcination zone within the pot expands, and its starting position shifts upward from L7 to the L5 region. Within the first 10 h of heating, the temperature response inside the pot is significant, and the initiation height of the high-temperature calcination zone approaches its steady-state range. After 15–20 h, the temperature field stabilizes, with temperatures in the L7 and L8 regions exceeding 1500 K, which may easily lead to coking and material blockage.

**Figure 6:**
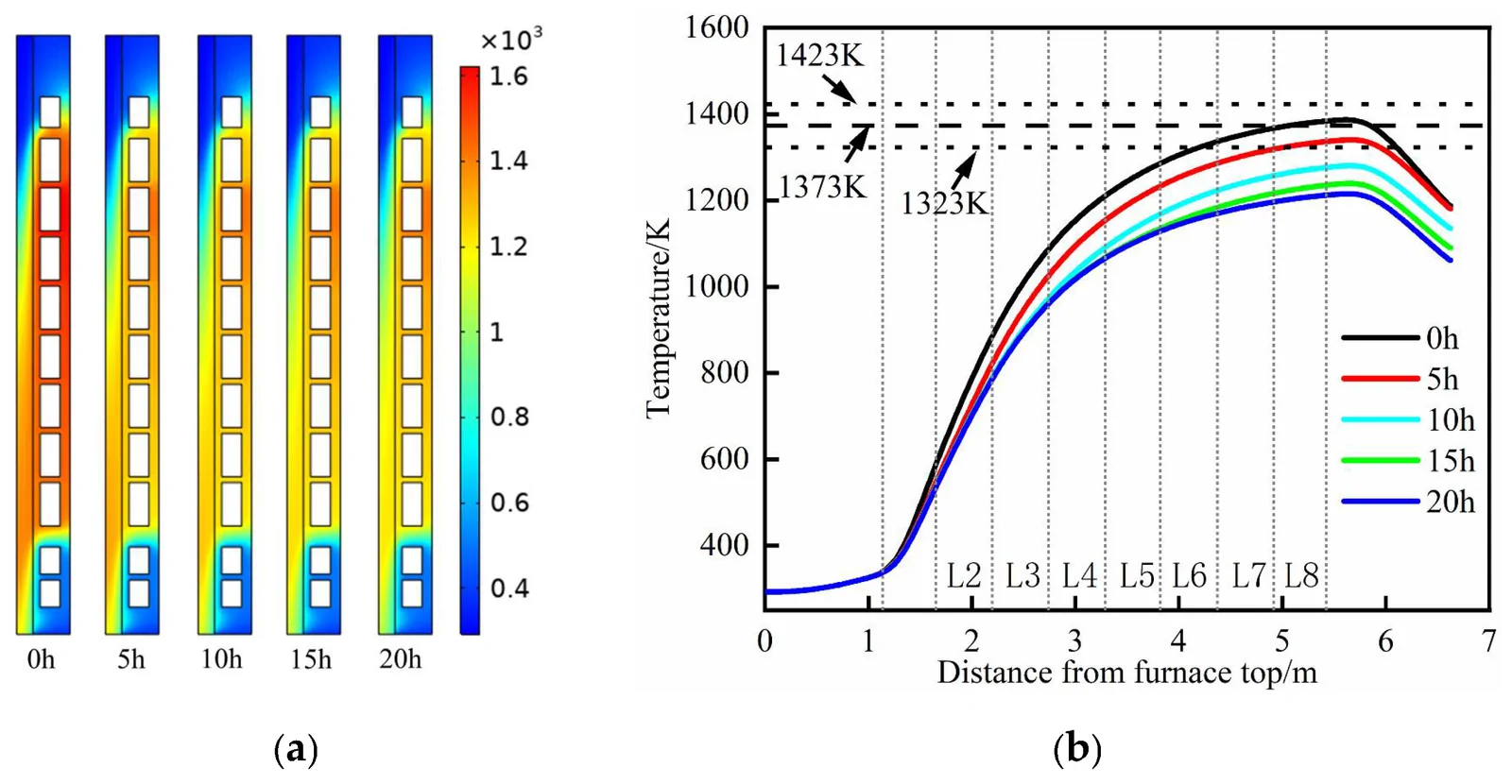
The temperature distribution of the model and the central temperature curve of the pot after the temperature of the flue decreases by 200 K: (**a**) model temperature distribution, (**b**) central temperature curve of pot.

Figure 6 presents the temperature contour and the temperature curves at the center of the pots after the flue temperature is decreased by 200 K from the baseline condition. As shown, when the flue temperature drops abruptly by 200 K, the flue wall temperature decreases rapidly within 5 h due to high thermal conductivity, approaching a steady state after about 10 h. The high-temperature calcination zone inside the pot shifts significantly downward. By 10 h, the maximum temperature within the pot falls below 1323 K, preventing the formation of an effective high-temperature calcination zone. Between 15 and 50 h, the temperature gradually stabilizes, but the overall temperature remains below 1200 K, which may easily lead to anomalies such as the presence of uncalcined material.

In summary, the distribution of the high-temperature calcination zone along the pot center is significantly affected by changes in the overall flue temperature and responds rapidly. In actual production, the distribution of the high-temperature calcination zone can be adjusted within a short period by controlling the overall flue temperature.

### 4.2. Impact of Upper Flue Temperature

The L1 and L2 flues are located in the upper region of the calciner and provide the prescribed thermal boundary conditions for coke heating. Step changes in their temperatures were imposed to investigate the resulting thermal response and calcination-zone migration.

**Figure 7:**
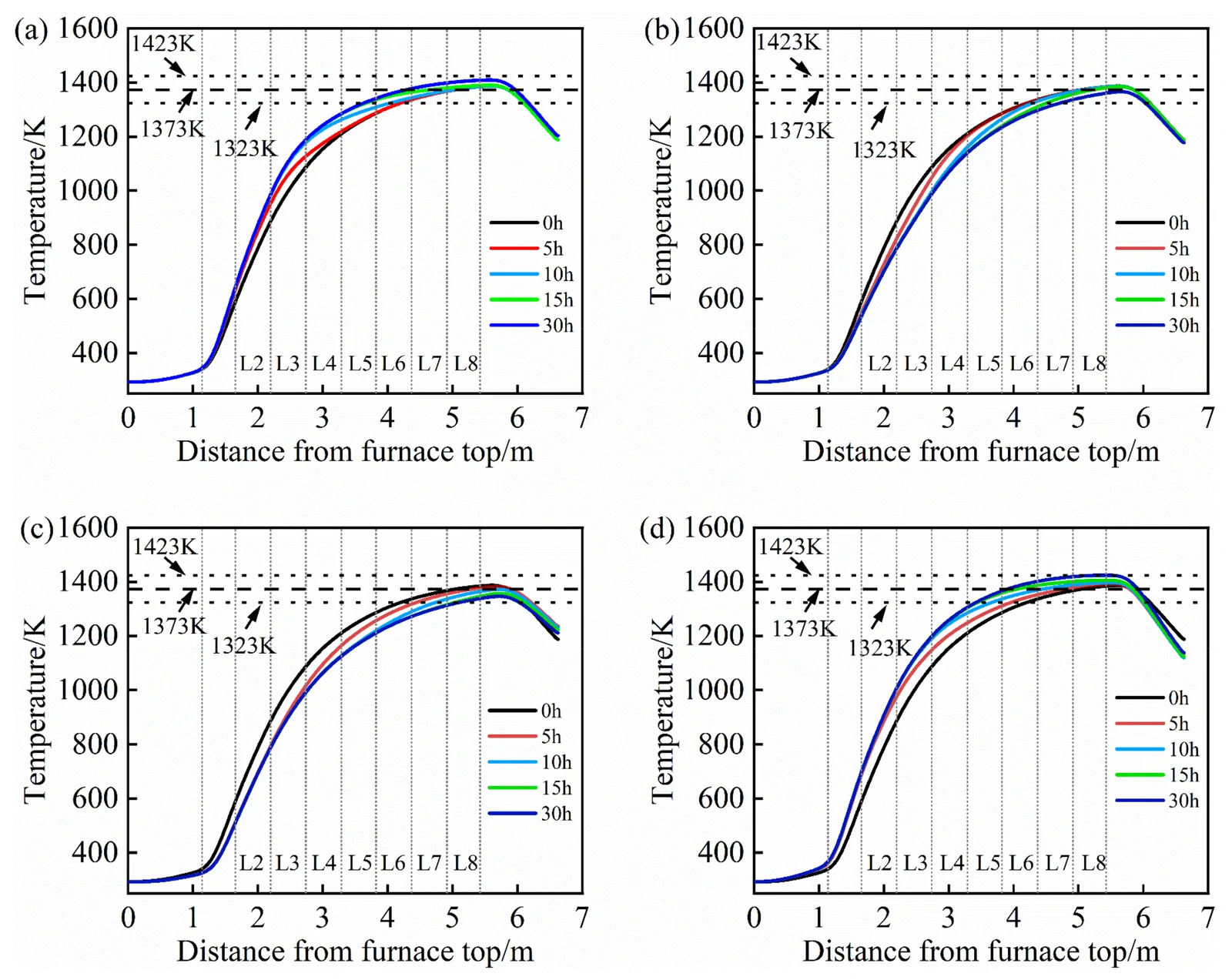
Time variation curve of the center temperature of the pot after sudden changes in different operating conditions: (**a**) the temperature of the upper flue suddenly increased by 200 K; (**b**) the temperature of the upper flue suddenly decreased by 200 K; (**c**) the unit discharge rate of a single pot suddenly increased from 95 kg/h to 115 kg/h; (**d**) the unit discharge rate of a single pot suddenly decreased from 95 kg/h to 75 kg/h.

After the baseline operating condition reached a steady state, the temperatures of the L1 and L2 flues were increased or decreased by 200 K. As shown in Figure 7a, following a 200 K increase, the overall pot-centerline temperature distribution changed relatively modestly, while the coke temperatures in the L3–L5 regions increased significantly. The enhanced heat transfer from the upper flues promoted coke heating and volatile release through the temperature-dependent pyrolysis kinetics. The high-temperature calcination zone extended from 1.3 to 1.9 m, and its upper boundary shifted to the upper part of L6 after 10 h and the middle of L5 after 15 h, with the migration rate gradually decreasing. The temperature field approached a new quasi-steady state after approximately 30 h.

As shown in Figure 7b, a 200 K decrease in the temperatures of the L1 and L2 flues primarily affected the L3–L5 regions. The reduced temperature difference between the flues and the coke bed weakened the thermal driving force for coke heating, resulting in lower coke temperatures in these regions. As the thermal disturbance propagated downward, the affected region extended to L6 after 10 h and L7 after 15 h. Meanwhile, the upper boundary of the high-temperature calcination zone gradually shifted downward, reaching the L7 region after 30 h, while its axial extent decreased from 1.3 to 0.75 m. This reduction in the high-temperature zone may increase the risk of insufficient coke calcination.

Overall, the results indicate that changes in upper-flue temperature significantly affect the axial position and extent of the high-temperature calcination zone. Appropriate adjustment of the upper-flue temperature may therefore provide a means of regulating the thermal state of the coke bed and mitigating the risks of over- and under-calcination.

### 4.3. Impact of Unit Discharge Rate

The unit discharge rate per pot refers to the amount of material discharged from a single pot per unit time during the calcination of petroleum coke in the VSC. This parameter is an important means of controlling furnace conditions in production.

After the standard operating condition stabilizes, we increased or decreased the unit discharge rate by 20 kg/h. As shown in Figure 7c, an increase in the unit discharge rate shortens the coke residence time. The heat supply fails to adjust synchronously, leading to a rapid temperature drop in the middle and lower regions. After 5 h, the high-temperature calcination zone begins to shift downward, moving to the L8 region within 20 h, and its length shortens from 1.3 m to 0.3 m, easily resulting in insufficiently calcined raw material. The temperature field approached a new quasi-steady state within approximately 15 h. Under the prescribed flue-temperature conditions, the increased discharge rate shortened the residence time of petroleum coke, resulting in lower coke temperatures and a shorter high-temperature calcination zone. As shown in Figure 7d, when the unit discharge rate decreases, the material residence time increases, heat accumulates in the upper part, and the temperature in the L2–L4 regions rise significantly, while the lower part heats slowly. Within 5–30 h, the starting position of the high-temperature calcination zone shifts upward from L6 to L5, and its length increases to 2 m. As the descent of petroleum coke slows, the L7 and L8 regions experience a slight temperature rise in the later stages, and the system eventually enters a new steady state.

In summary, adjustments in the unit discharge rate significantly affect the temperature distribution and the position of the high-temperature calcination zone. Changes in the unit discharge rate influence the descent speed of petroleum coke and the time to reach steady state. However, within 5 h, the affected area of the temperature at the center of the pot is consistently the L2 to L4 region. This indicates that in the short term, the regulatory effects of natural heat diffusion and convection on temperature distribution are consistent.

## 5. Machine Learning Algorithm Training, Prediction, and Evaluation

Accurate prediction of the transient temperature distribution along the pot centerline is important for assessing the thermal response of vertical shaft calciners to changes in operating conditions. However, the computational cost of transient numerical simulations limits their suitability for rapid temperature prediction. Therefore, TCLCA was developed to predict the spatiotemporal evolution of centerline temperature under the evaluated flue-temperature disturbance conditions. By integrating temporal and spatial feature extraction, the model captures the thermal-response delay and spatial coupling within the calcination zone. It provides accurate predictions at substantially lower computational cost than numerical simulation, supporting rapid thermal-state evaluation and operating adjustment.

### 5.1. Temporal Prediction Concept

For the task of predicting the temporal evolution of temperature at the center of the pot, observation points along the centerline are used to construct temperature time series. These series characterize the temperature variation over time at each point under different operating conditions. The temporal prediction problem for the centerline temperature can be defined as follows:

*X_k_* = *A_t_*_−*k*_, *A_t_*_−*k*+1_,…, *A_t_*_−1_ (8)

*Y_l_* = (*A_t_*, *A_t_*_+1_,…, *A_t_*_+*l*−1_) (9)

(*A_t_*, *A_t_*_+1_,…, *A_t_*_+*l*−1_) = *f*(*A_t_*_−*k*_, *A_t_*_−*k*+1_,…, *A_t_*_−1_) (10)

where *X* denotes the historical temperature input sequence, *A_t_* is the centerline temperature vector at time step t, and *Y* is the corresponding prediction target. The parameters *k* and *l* denote the historical input length and prediction horizon, respectively.

The TCLCA model uses historical temperature profiles at 510 locations along the pot centerline to predict the temperature profile at the next time step. Flue temperature and per-pot discharge rate are not explicitly included as model inputs. The machine-learning dataset consists of numerically generated temperature sequences under nine flue-temperature disturbance conditions. Therefore, the demonstrated predictive capability is limited to the corresponding temperature evolution, and the model has not been validated for independent changes in discharge rate.

**Figure 8:**
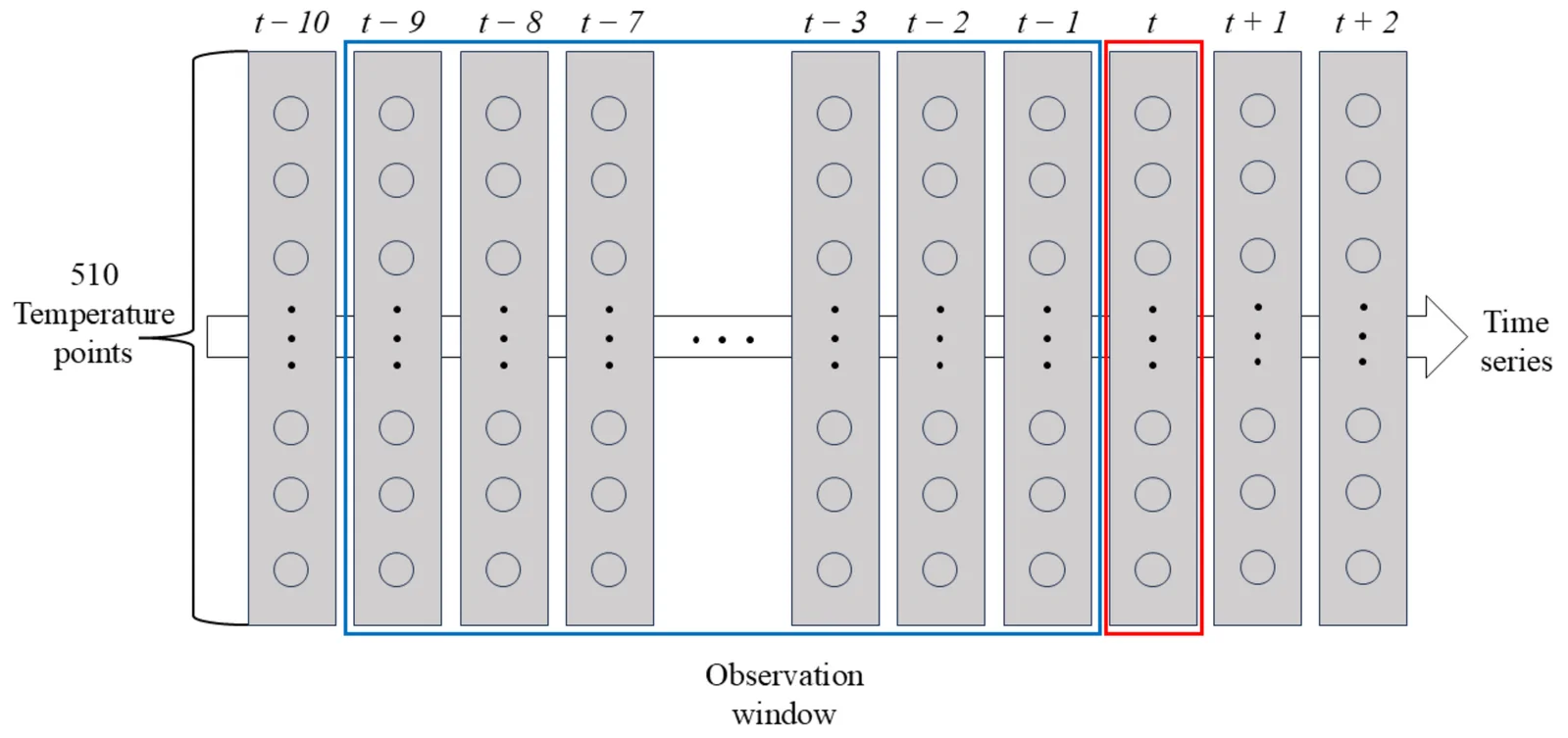
Time Series and Sliding Window of Temperature at the Center of the Pot (ellipsis indicates omitted time steps).

We predicted the pot-centerline temperature by converting the one-dimensional temperature sequence into a two-dimensional matrix through the sliding window method. The historical sequence and the sequence to be predicted are respectively set as the observation window and the prediction window. The observation window contains the temperature data of *k* consecutive historical time steps, and the prediction window contains the temperature value of the next time step. After the prediction is completed, the two windows slide forward by one time step to generate the next set of samples until the end of the sequence. As shown in Figure 8, in this study, the historical input window contains nine consecutive temperature profiles, which are used to predict the temperature profile at the next time step. Thus, each supervised sample consists of nine input time steps and one target time step, giving a total sequence length of 10. The historical input length and total sample length are distinguished accordingly.

**Figure 9:**
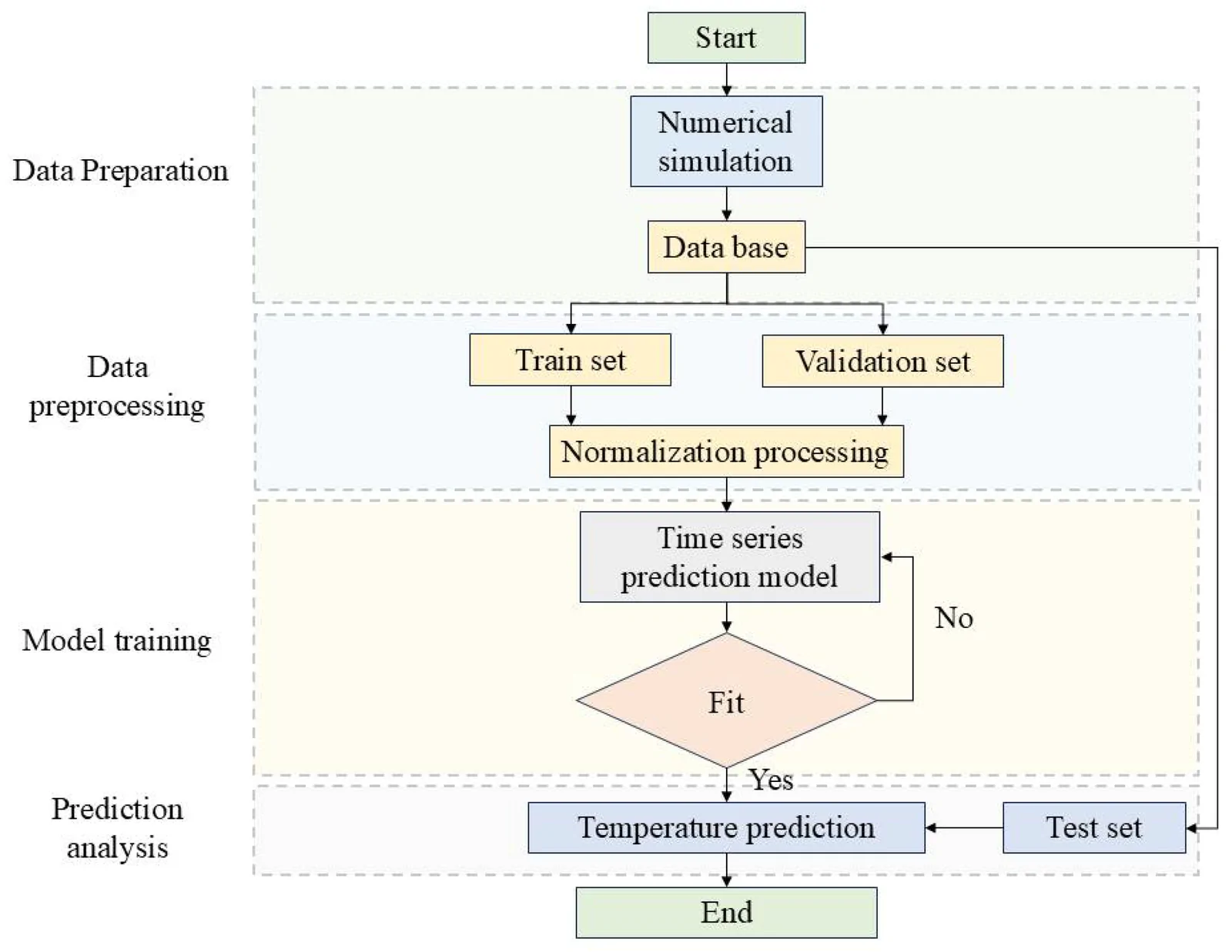
Workflow of simulation data-driven modeling.

The construction of the prediction model mainly includes four different stages: data preparation, data preprocessing, model training, and prediction analysis. Figure 9 shows the workflow and methods adopted in this paper. 

In the model training stage, the TCLCA model is used to train the training set, and the impact of different architectures on prediction accuracy is compared. The network is adjusted based on the prediction results obtained from the validation set to optimize its performance. The trained model is used to perform single-step and recursive predictions on the test set, and the prediction results are compared with the simulation results to analyze the prediction ability of the model.

### 5.2. Construction of the Temperature Dataset

#### 5.2.1. Data Preparation

The annual calcination data of a certain petroleum coke smelting plant is collected and statistically analyzed. The temperature of the flues and the unit discharge rate of each pot throughout the year is analyzed. The fluctuation range of the L2 flue temperature is from 1100 K to 1580 K, the fluctuation range of the eight-layer flue temperature is from 950 K to 1300 K, and the variation range of the unit discharge rate of each pot is between 45 kg/h and 120 kg/h.

The two-dimensional pot simulation model is divided into 510 × 45 cells of approximately the same size through the square processing of quadrilateral cells. Each cell is a temperature monitoring point, and the temperature value of each point changes over time. The center temperature of the pot can be represented by a 510 × 1 one-dimensional matrix, and a time series of the center temperature of the pot is generated over time. The one-dimensional temperature matrix is composed into a two-dimensional temperature matrix through the observation window as the input to predict the center temperature of the pot at the next moment. The specific operation is shown in Figure 8.

Based on the transient thermal-response analysis, nine flue-temperature disturbance conditions were simulated to generate the temperature dataset. Taking the L2 flue as an example, its temperature was changed from the baseline value of approximately 1623 K to 1423, 1473, 1523, 1573, 1673, 1723, 1773, 1823, and 1873 K. Seven conditions were used for training, while the 1823 K and 1873 K conditions were reserved for validation and independent testing, respectively. The datasets were divided by operating condition rather than by individual time windows. For each condition, 100 centerline temperature profiles, each containing 510 spatial points, were sampled at 0.2 h intervals from 0 to 19.8 h, yielding a total of 4.59 × 10^5^ temperature values.

#### 5.2.2. Data Preprocessing

The numerically generated centerline temperature data were preprocessed before model training. The nine operating conditions were first divided into training, validation, and test sets. Min–Max scaling parameters were determined independently for each of the 510 centerline locations using only the training input sequences. The same parameters were subsequently applied to the training targets and the validation and test datasets without refitting, thereby preventing information leakage from the held-out conditions. The fitted scaling parameters were also used to transform the predicted temperatures back into physical units for performance evaluation. The Min–Max transformation is expressed as follows:

(11) $$x^{\prime }=\frac{x-\mathrm{Min}}{\mathrm{Max}-\mathrm{Min}}$$

where x and x′ denote the original and normalized temperature values, respectively; *Min* and *Max* represent the minimum and maximum temperature values at the corresponding centerline location, determined exclusively from the training input sequences.

#### 5.2.3. Model Evaluation Indicators

To evaluate the prediction performance, we adopted the following three typical evaluation indicators.

(12) $$\mathrm{MSE}=\frac{1}{n}\sum_{i=1}^{n}{\left(y_{i}-y_{i}^{*}\right)}^{2}$$

(13) $$\mathrm{MAE}=\frac{1}{n}\sum_{i=1}^{n}|y_{i}-y_{i}^{*}|$$

(14) $$R^{2}=1-\frac{\sum_{i=1}^{n}{\left(y_{i}-y_{i}^{*}\right)}^{2}}{\sum_{i=1}^{n}{\left(y_{i}-{\bar{y}}_{i}\right)}^{2}}$$

where n represents the number of samples, y*_i_* is the true value of the i-th sample, $y_{i}^{*}$ is the predicted value of the i-th sample, and ${\bar{y}}_{i}$ is the average of the true values.

### 5.3. Model Architecture

**Figure 10:**
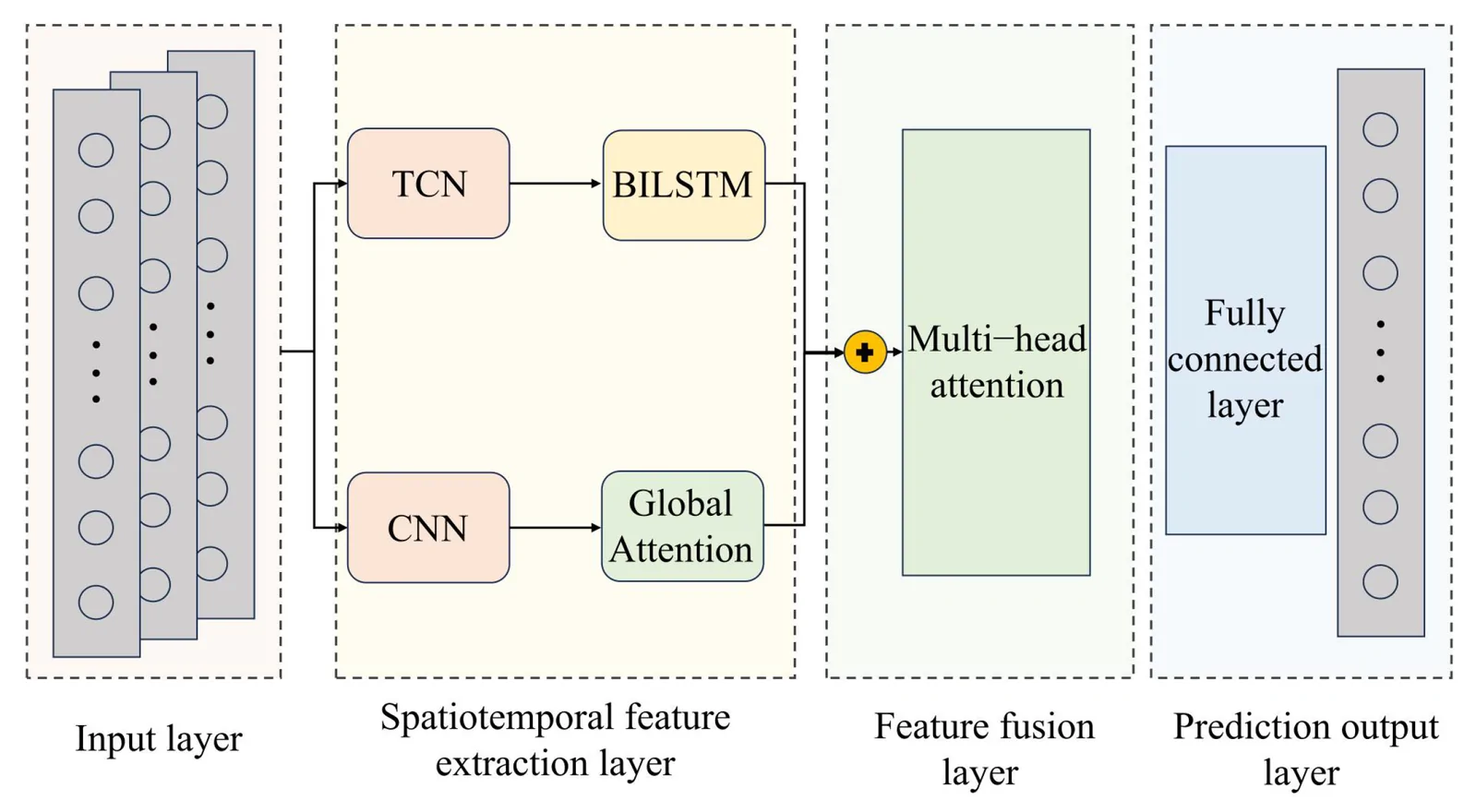
Architecture of the dual-channel spatiotemporal feature fusion temperature prediction model.

The dual-channel spatiotemporal feature fusion pot-centerline temperature prediction model TCLCA introduced in this paper is mainly divided into four modules, including the TCN-BiLSTM layer, the CNN-Attention layer, the feature fusion layer, and the temperature prediction output layer. The overall model is shown in Figure 10:

#### 5.3.1. TCN-BiLSTM

**Figure 11:**
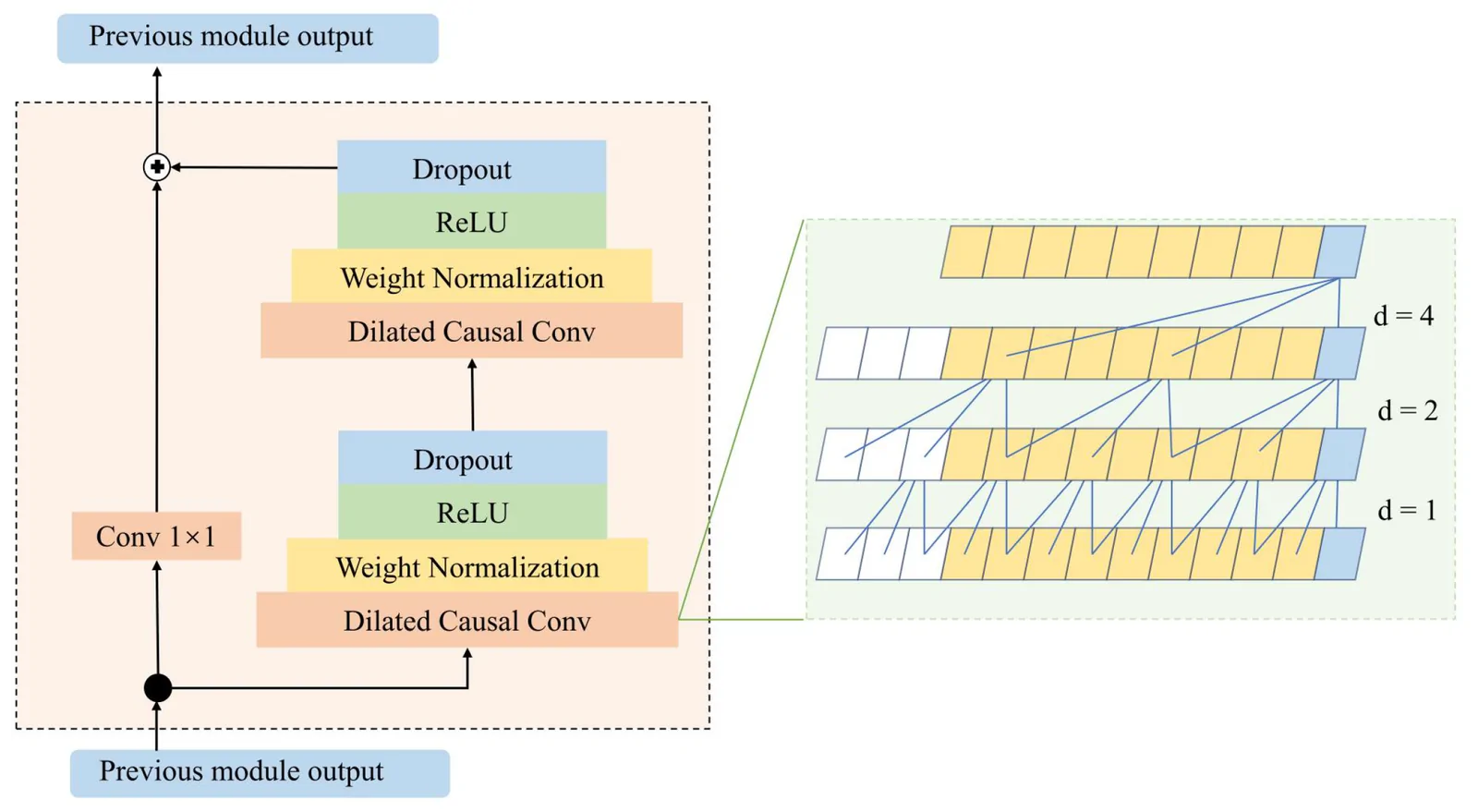
Schematic diagram of the TCN structure.

The TCN-BiLSTM layer combines the Temporal Convolutional Network (TCN) and BiLSTM to fully leverage the advantages of both, making the model more robust. The structure of TCN is shown in Figure 11.

TCN introduces dilated convolution, which inserts intervals between the elements of the convolution kernel, causing the receptive field of each layer of convolution to expand exponentially, thereby effectively capturing the long-term dependencies of the input data. For a given input sequence and convolution kernel, the output *y*_t_ at time t of dilated convolution satisfies:

(15) $$y_{t}=\sum_{i=0}^{k-1}f_{i}x_{t-d\cdot i}$$

where *k* is the size of the convolution kernel, and d is the dilation rate, which determines the actual spacing between the elements of the convolution kernel.

For a given input *X_TCN_* and convolution operation *F*(*x*), the output of the residual connection can be expressed as:

*H*(*X_TCN_*) = *F*(*X_TCN_*) + *X_TCN_* (16)

where F(x) is the output of the convolutional layer or multiple convolution operations and nonlinear activation functions, and x is the original input.

To enhance the model’s ability to extract bidirectional hidden features at different time scales, the output h of the TCN is used as the input to the BiLSTM. The formula is as follows:

(17) $$\vec{h_{t}}=\mathrm{LSTM}(\vec{x_{t}},\vec{h_{t-1}})$$

(18) $$\vec{h_{t}}=\mathrm{LSTM}(\vec{x_{t}},\vec{h_{t+1}})$$

(19) $$H_{t}=[\vec{h_{t}}⊕\vec{h_{t}}]$$

where $\vec{h_{t}}$ and $\vec{h_{t}}$ are the outputs of the forward and backward LSTM at time t, respectively, $\vec{h_{t}}$ is the input at time t, and ⊕ denotes the vector concatenation operation.

By combining TCN and BiLSTM, the model integrates multi-scale temporal feature extraction with bidirectional sequence modeling to capture complex temperature evolution patterns.

#### 5.3.2. CNN-Attention

The CNN-Attention layer primarily consists of two core components: a multi-channel residual CNN module and a global attention module. In the prediction of the pot temperature, the temperature at different points is jointly influenced by local heat conduction and the global ambient temperature. The multi-channel design enables the model to simultaneously focus on local temperature fluctuations and spatial correlations over a larger range, thereby enhancing the comprehensiveness of feature representation.

Residual connections directly add the input to the convolutional output. Meanwhile, 1 × 1 convolutions align channel dimensions. This ensures consistent feature map shapes throughout the network.

The formula for the residual connection is as follows:

*Y* = *F*(*X_CNN_*) + *X_CNN_* (20)

where *Y* is the input to the next layer, *F*(*X*) is the output after the convolution operation, and *X_CNN_* is the input to the current layer. If the dimensions of the input and output do not match, a 1 × 1 convolution is applied to *X_CNN_* for a linear transformation to align the dimensions. The application of residual connections effectively mitigates the vanishing gradient problem in the network, ensuring stable gradient propagation.

The Global Attention Mechanism aims to capture global spatial dependencies by weighting the features at all positions. In the heat conduction process of the petroleum coke pot, the evolution of the temperature field, induced by the settling of petroleum coke, exhibits significant spatial and temporal hysteresis. For a specific temperature measurement point on the centerline of the pot, its current temperature is influenced by the temperatures at points above it at previous times. The global attention mechanism dynamically adjusts the weights of different positions, highlighting the spatial features most critical for predicting the target, thereby enhancing the model’s ability to understand the global temperature distribution.

The output of the multi-channel residual CNN module is weighted by the global attention mechanism to form the final spatial feature representation of the temperature evolution over time at the center of the VSC pot. This process achieves effective fusion of local and global features.

#### 5.3.3. Feature Fusion

The feature fusion layer integrates temporal and spatial features extracted by the TCN-BiLSTM and CNN-Attention layers. This integration enables the model to capture both local and global information in temperature time-series data effectively.

The Multi-Head Attention mechanism is a core component of the Transformer model. It is based on the self-attention principle. The mechanism involves three main steps. First, input features are linearly transformed into three vectors: Query (*Q*), Key (*K*), and Value (*V*). Second, scaled dot-product attention calculates the attention weights for each head. Finally, outputs from all heads are concatenated and linearly transformed into the final representation.

For the i-th attention head, the attention score is computed as follows:

(21) $$\mathrm{Attention}(Q_{i},K_{i},V_{i})=\mathrm{softmax}\left(\frac{Q_{i}K_{i}^{T}}{\sqrt{d_{k}}}\right)V_{i}$$

where $\sqrt{d_{k}}$ is a scaling factor. It reduces numerical instability in high-dimensional dot products.

Each attention head independently focuses on different feature subspaces. This allows multi-perspective analysis of the input. The feature fusion calculation is defined as:

(22) $${\mathrm{Head}}_{i}=\mathrm{Attention}(QW_{i}^{Q},KW_{i}^{K},VW_{i}^{V})$$

*x* = *Concat*(*head*_1_,…, *head_k_*)*W* (23)

Multi-head attention offers advantages in parallel computation and multi-view feature extraction. It dynamically assigns weights to adaptively fuse local temporal patterns with global spatial thermal relationships. This enhances the integration of spatiotemporal features and improves the model’s sensitivity to critical information.

#### 5.3.4. Temperature Prediction

The fused features are flattened and fed into the prediction output layer. Dropout regularization is applied to enhance generalization and prevent overfitting. A fully connected layer then performs linear mapping on the fused features. This produces the predicted temperature profile at all 510 locations along the pot centerline.

### 5.4. Experimental Training

#### 5.4.1. Experimental Setup

The model was trained under the following environment: GPU: GTX 1050; OS: Windows 11 64-bit; Programming Language: Python 3.8; Framework: TensorFlow 2.8.2; CUDA Version: 11.1. Specific model hyperparameters are listed in Table 6.

The Adam optimizer was used throughout the experiments. The cosine annealing scheduler was applied to adjust the learning rate periodically via a cosine function [27]. This approach improves the model’s training effectiveness.

**Figure 12:**
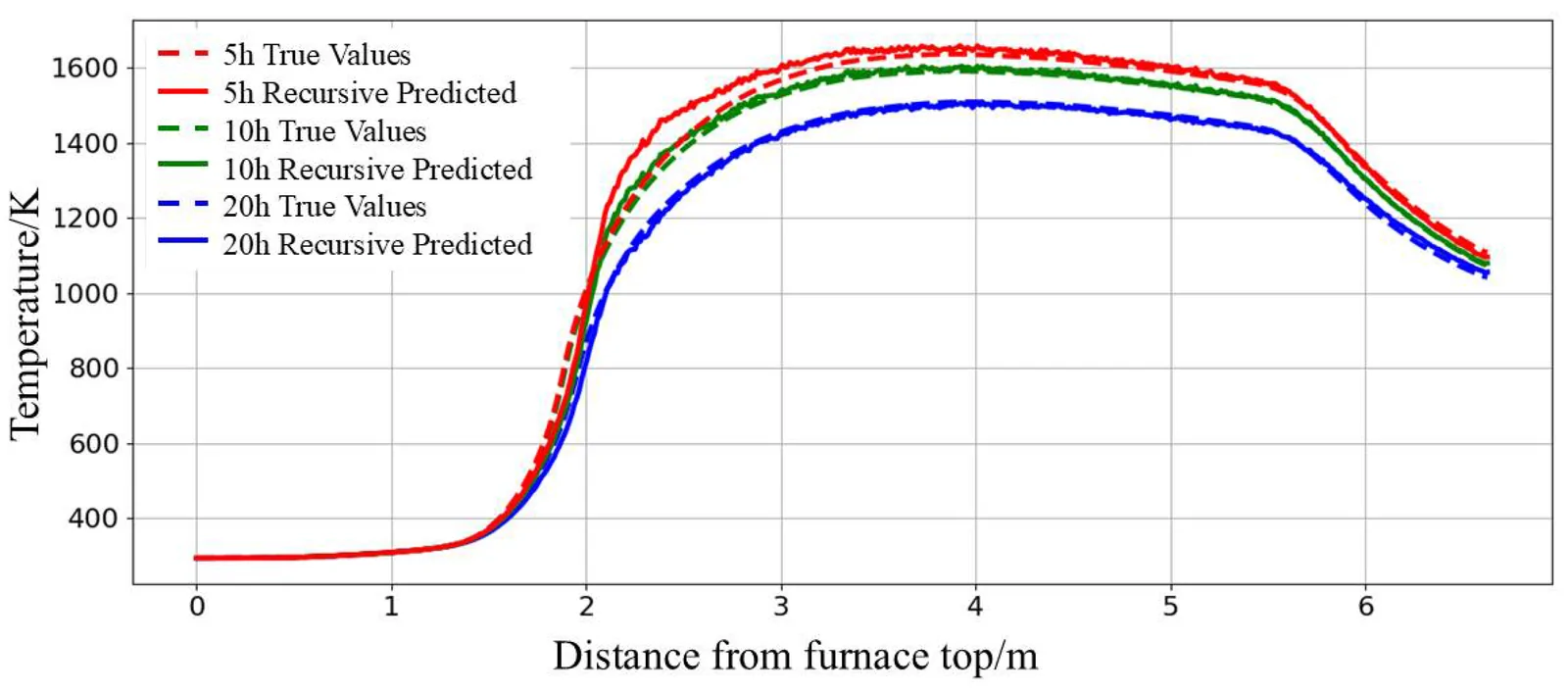
Comparison of single-step prediction results.

**Figure 13:**
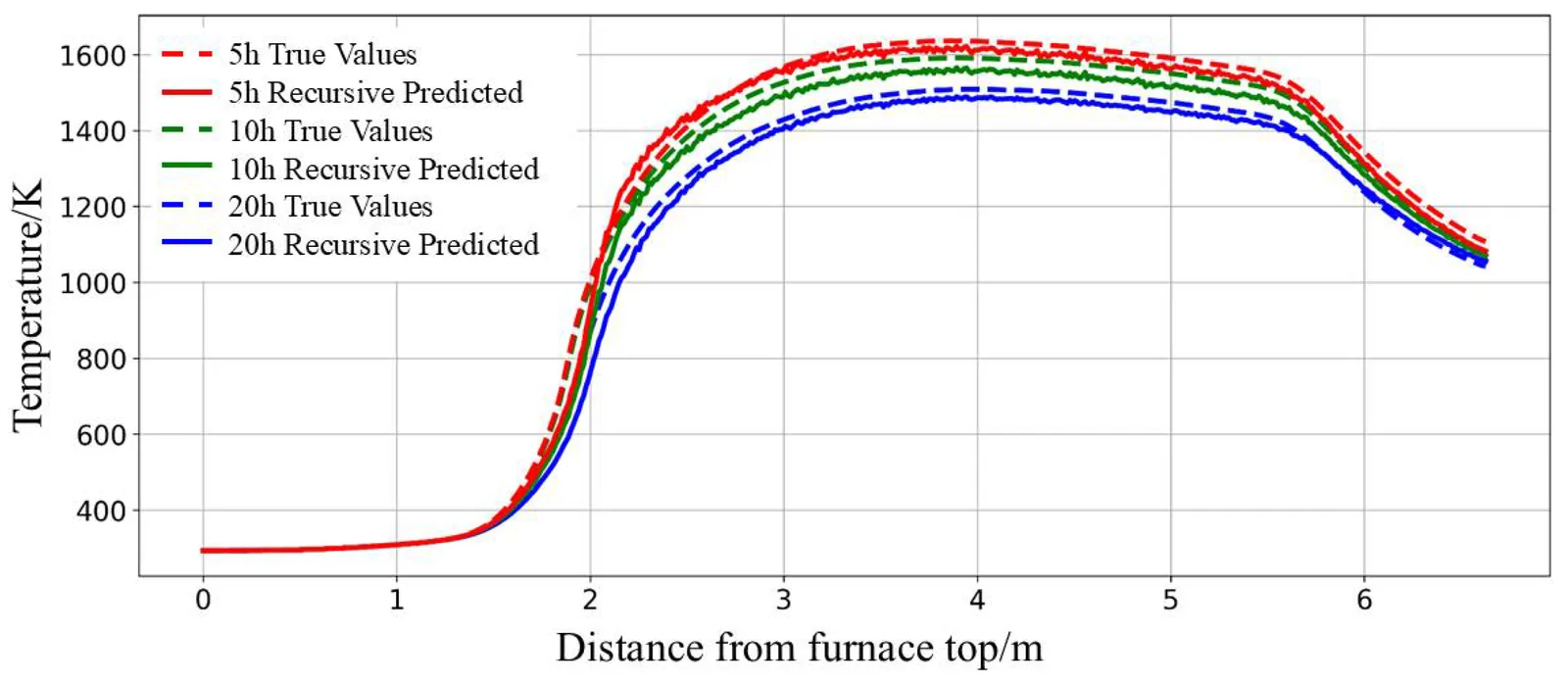
Comparison of recursive prediction results.

The TCLCA model was tested under working conditions using single-step and recursive prediction. This procedure validated its prediction performance relative to simulation models. The results are shown in Figure 12 and Figure 13.

In single-step prediction, the model uses the preceding nine centerline temperature profiles to predict the temperature profile at the next time step. Recursive prediction was initialized using the first nine Numerically generated centerline temperature profiles of the test condition. The model then generated 91 consecutive temperature profiles by feeding each predicted profile back into the input window, without using additional simulation temperature values during the recursive process. These predictions were compared with the corresponding numerically generated profiles within the available simulation interval. Therefore, the reported results assess recursive prediction accuracy and error accumulation within the simulated time range, rather than temporal extrapolation beyond that range.

The single-step predictions closely match the actual simulated values. This indicates excellent short-term prediction capability. The recursive prediction results align well with the true curve in high-temperature calcination and low-temperature zones. A slight deviation appears near the furnace top toward the end of the prediction period. Overall, the prediction error remained limited over the evaluated recursive sequence, indicating satisfactory predictive accuracy under the held-out test condition.

For the evaluated centerline temperature sequence covering a physical simulation period of 0–1200 min, the numerical simulation required 161 s of computational time, whereas TCLCA recursive inference required approximately 10 s for the corresponding prediction task. This represents a reduction in computational time of approximately 94%.

#### 5.4.2. Comparative Experiments

Comparative experiments evaluated the TCLCA model against other time series forecasting models. Performance was measured using MAE, MSE, and *R*^2^ metrics. Results are provided in Table 7 and Table 8.

The following models were compared:

(1) FC-LSTM [28]: This model uses an LSTM network to capture temporal dependencies. A fully connected layer produces the final prediction.
(2) Transformer: The Transformer employs multi-head attention and is widely used in time series tasks.
(3) LSTNet [29]: This model combines CNN and LSTM to extract short-term local dependencies and long-term trends. An autoregressive component addresses scale insensitivity.
(4) DGCnetwork [30]: A three-layer GRU extracts temporal features. A CNN captures spatial features. A fully connected layer integrates these features for prediction.

Under the evaluated test condition, TCLCA outperformed the comparison models in both single-step and recursive prediction, demonstrating its effectiveness for the investigated temperature-prediction task.

#### 5.4.3. Ablation Experiments

Ablation experiments were conducted to evaluate the impact of each component in the TCLCA model on temperature prediction performance. The results are shown in Table 9 and Table 10. Evaluation metrics include MSE, MAE, and *R*^2^.

Four variant models were designed:

(1) TCLCA-TCN variant: The TCN module was removed from the temporal feature channel. Only BiLSTM was used for temporal feature extraction.
(2) TCLCA-BiLSTM variant: The BiLSTM module was removed from the temporal feature channel. Only the multi-scale dilated TCN was used for feature extraction.
(3) TCLCA-CNN variant: The CNN-Attention layer was removed from the spatial feature extraction branch. The model relied solely on the TCN-BiLSTM layer.
(4) TCLCA-Attention variant: The multi-head attention mechanism was removed from the feature fusion process. Simple concatenation was used instead.

Experimental results show that the complete TCLCA model outperforms all ablation variants across all metrics. Removing the TCN module weakened the model’s ability to capture temporal features. Removing BiLSTM limited the model’s capacity for modeling bidirectional temporal relationships. Removing the CNN module significantly reduced the model’s ability to model spatial information. Removing the multi-head attention mechanism led to decreased performance and larger prediction deviations. This confirms the advantage of the attention mechanism in integrating spatiotemporal features.

The TCLCA model maintains low error accumulation and high fitting accuracy in long-term recursive prediction. All ablation variants show performance degradation. Removing the TCN module weakens long-term temporal feature capture, causing a slight error increase. Removing the BiLSTM causes significant performance deterioration. This confirms its important role in modeling temporal dependencies and long-term trends. Compared with single-step prediction, the CNN-Attention layer shows smaller error accumulation in recursive prediction. The absence of spatial features has less impact in long-term recursive scenarios. Removing the multi-head attention mechanism causes a sharp error increase and significant *R*^2^ drop. This suggests an important contribution in feature fusion for long-term recursive prediction.

As TCLCA was trained exclusively on numerically generated temperature data, its predictive accuracy is inherently dependent on the reliability of the underlying numerical model. The discrepancies observed in the L3–L5 regions (Figure 3) may therefore propagate to the surrogate predictions. Accordingly, the reported prediction accuracy reflects agreement with numerical results rather than direct validation against actual furnace temperatures.

The TCLCA model achieved satisfactory predictive accuracy under the held-out test condition. Further evaluation under additional operating conditions would provide a more comprehensive assessment of its generalizability.

## 6. Conclusions

A transient two-dimensional multiphysics simulation model was developed to investigate the thermal response of a petroleum coke vertical shaft calciner under changes in flue temperature and per-pot discharge rate. The results showed that increasing the flue temperature shifted the high-temperature calcination zone upward and increased its axial extent, whereas increasing the discharge rate shortened the residence time of petroleum coke and displaced the high-temperature zone downward, thereby increasing the risk of insufficient calcination. Following a step change in operating conditions, the furnace wall approached a quasi-steady thermal state within approximately 10 h, while the coke bed exhibited a slower thermal response and required up to approximately 40 h to reach a quasi-steady temperature distribution.

Based on the numerical simulations, 4.59 × 10^5^ spatiotemporal temperature values from 510 centerline locations under nine flue-temperature disturbance conditions were constructed as a dataset for surrogate modeling. A hybrid machine learning surrogate, TCLCA, was developed to predict the transient evolution of centerline temperature. In recursive prediction, the surrogate achieved an *R*^2^ of 0.997 and an MAE of 18.69 K over the evaluated transient sequence. For the same centerline temperature sequence, the calculation time was reduced from 161 s using the numerical model to approximately 10 s using surrogate-model inference, corresponding to a reduction of about 94% under the reported computational conditions.

Overall, the proposed simulation-driven surrogate framework provides an efficient approach for rapidly predicting transient temperature evolution and assessing calcination-zone migration in petroleum coke vertical shaft calciners. The numerical model provides insight into the thermal responses to flue-temperature and discharge-rate disturbances, while TCLCA enables rapid prediction of centerline temperature evolution under the evaluated flue-temperature disturbance conditions.

## Figures and Tables

**Table 1:** Comparison of previous studies with the present work.

Application	Simulation Method/ML Model	Inputs/Outputs	ML Dataset Size	Difference from the Present Study
VSC [2]	Real-time 2D mathematical model	Input: Operating parameters; Output: 2D temperature field	N/A	Physics-based rapid temperature prediction
10-layer pot calciner [17]	3D fluid–solid coupled CFD model	Input: Operating boundary conditions; Outputs: Flow, pressure, and temperature fields	N/A	Flow and thermal distributions under operating conditions
24-pot calciner [18]	Two-fluid CFD model	Input: Operating conditions; Outputs: Temperature, moisture, volatile, and velocity fields	N/A	Detailed calcination-process simulation
VSC [19]	3D two-fluid model	Inputs: Discharge rate, volatile content, excess air; Outputs: Calcination-zone position and length	N/A	Parameter-dependent calcination-zone migration
56-pot, 8-layer VSC [20]	3D unsteady two-fluid CFD	Inputs: Volatile inlet configuration, excess air, outlet pressure; Outputs: Flow and temperature fields	N/A	Unsteady CFD and operating-parameter analysis
Rotary kiln [21]	CFD + GA-RF	Inputs: Operating conditions and material properties; Output: Temperature field	625 CFD cases	Temperature prediction in a different furnace type
Blast furnace [22]	Mechanistic model and ensemble ML	Inputs: Injection parameters and spatial coordinates; Outputs: Internal-state fields	270 cases; 6.43 × 10^6^ spatial samples	Spatial internal-state prediction
Vertical annealing furnace [23]	3D FEM and RF	Inputs: Wafer spacing and heater configuration; Output: Temperature uniformity	300 simulation samples	Temperature-uniformity optimization
24-pot, 8-layer VSC (representative pot)	2D transient simulation and TCLCA	inputs: Flue temperature and discharge rate; outputs: Transient temperature field and calcination-zone migration; ML input: Historical centerline temperatures; ML output: Next-step centerline temperatures	9 disturbance cases; 4.59 × 10^5^ temperature values	Transient step-response analysis combined with recursive temperature prediction

Note: N/A indicates that no machine-learning model was employed in the corresponding study.

**Table 2:** Physical property parameters of petroleum coke and furnace lining materials.

Material	Density, *⍴* (kg∙m^−3^)	Effective Heat capacity, C_p_ (J·kg^−1^∙K^−1^)	Thermal Conductivity, λ (W∙m^−1^∙K^−1^)	Void Fraction of the Packed Petroleum Coke *ε*_*s*_ (-)	Reference
Petroleum coke	1700	97.35 + 26.2 × T	(−4.084 + 2.479 × 10^−2^ T) × (d_p_)^0.464^	0.58 × (100d_p_)^0.1^	[24,25]
Silica brick	1925	1093	1.05 + 9.3 × 10^−4^ T	-	[17]

**Table 3:** Mass fractions and reaction rates of various substances in petroleum coke pyrolysis.

Pseudo-Component	Mass Fraction	Reaction Rate, s^−1^	Reference
1	0.41	1096.63 × (1 − *ξ*)^1.66^ × exp(−92.36 × 10^3^/(8.314 × *T*))	[25]
2	0.23	1096.63 × (1 − *ξ*)^1.66^ × exp(−73.52 × 10^3^/(8.314 × *T*))	[25]
3	0.36	1096.63 × (1 − *ξ*)^3.26^ × exp(−111.15 × 10^3^/(8.314 × *T*))	[25]

**Table 4:** Initial and boundary conditions of the baseline numerical model.

Parameters	Unit	Values
Initial temperature field	K	T_ss_(x,y)
Initial species concentration fields	-	c_i,ss_(x,y), i = 1, 2, 3
Petroleum coke inlet temperature	K	293
Per-pot discharge rate	kg·h^−1^	95
Flue temperatures (L1–L8)	K	1473.2, 1623.2, 1573.2, 1523.2, 1503.2, 1473.2, 1453.2, 1423.2
Flue inlet temperature	K	373
Initial mass fractions of volatile pseudo-components in the petroleum coke at the inlet	-	c_1,0_ = 0.41, c_2,0_ = 0.23, c_3,0_ = 0.36
Thermal outlet	W·m^−2^	−**n** · **q** = 0
Symmetry and insulated boundaries	W·m^−2^	−**n** · **q** = 0
Species no-flux boundaries	kg/(m^2^·s)	−**n** · **j**_i_ = 0

**Table 5:** Two-dimensional geometric parameters of the vertical shaft calciner.

Parameter Name	Value
Height of pot	6.627 m
Width of pot	0.18 m
Length of pot	1.66 m
Thickness of furnace wall	0.409 m
Thickness of flue	0.219 m
Height of flue interval	0.065 m

**Table 6:** Hyperparameter Settings.

Parameter	Value
epochs	200
patience	20
batch	16
lr	0.0001
Historical input length	9
Prediction horizon	1
Total sequence length	10

**Table 7:** Performance comparison of TCLCA model with other time series prediction models in single-step prediction.

Model	MSE (Normalized)	MAE (Normalized)	*R* ^2^
FC-LSTM	0.0187	0.0862	0.9974
Transformer	0.0104	0.0617	0.9981
LSTNet	0.0148	0.0672	0.9980
DGCnetwork	0.0162	0.0864	0.9971
TCLCA	0.0088	0.0538	0.9986

**Table 8:** Performance comparison of TCLCA model with other time series prediction models in recursive prediction.

Model	MSE (K^2^)	MAE (K)	*R* ^2^
FC-LSTM	10,056.3923	76.8593	0.9608
Transformer	1012.8434	21.4567	0.9837
LSTNet	7384.5594	68.1689	0.9723
DGCnetwork	7084.2267	65.5653	0.9724
TCLCA	760.6533	18.6888	0.9970

**Table 9:** Results of ablation experiments for TCLCA model.

Model	MSE (Normalized)	MAE (Normalized)	*R* ^2^
TCLCA-TCN	0.0128	0.0708	0.9978
TCLCA-BiLSTM	0.0130	0.0679	0.9983
TCLCA-CNN	0.0189	0.0892	0.9969
TCLCA-Attention	0.0145	0.0713	0.9981
TCLCA	0.0088	0.0538	0.9986

**Table 10:** Recursive prediction results of different variants in TCLCA model ablation experiments.

Model	MSE (K^2^)	MAE (K)	*R* ^2^
TCLCA-TCN	7398.4624	65.0351	0.9712
TCLCA-BiLSTM	16,825.9506	95.6531	0.9345
TCLCA-CNN	8793.4323	68.5235	0.9658
TCLCA-Attention	48,996.5712	162.3139	0.8093
TCLCA	760.6533	18.6888	0.9970

## Data Availability

The numerically generated temperature data and machine-learning prediction results supporting the findings of this study are available from the corresponding author upon reasonable request.

## Abbreviations

Nomenclature Symbols		*v_s_* *_0_*	Initial longitudinal solid-phase velocity (m/s)
		*u_s_* *_0_*	Initial transverse solid-phase velocity (m/s)
VSC	Vertical shaft calciner	*Q*_m_	Mass flow rate of raw coke (kg/s)
TCLCA	Temporal Convolutional Network–Bidirectional Long Short-Term Memory–Convolutional Neural Network–Attention	*A*_c_	Cross-sectional area of the pot (m^2^)
CFD	Computational Fluid Dynamics	*C*_p_	Effective specific heat capacity (J·kg^−1^·K^−1^)
TCN	Temporal Convolutional Network	*K*_eff_	Effective thermal conductivity (W·m^−1^·K^−1^)
BiLSTM	Bidirectional Long Short-Term Memory	*S*_i_	corresponding reaction source term (kg·m^−3^·s^−1^)
CNN	Convolutional Neural Network	*d*_p_	Equivalent particle size of petroleum coke (m)
LSTM	Long Short-Term Memory	*Y*	Input of the residual connection (–)
GRU	Gated Recurrent Unit	*T*	Temperature (K)
FC-LSTM	Fully Connected LSTM	*t*	Time (s or h)
LSTNet	Long- and Short-Term Time-series Network	*n*	Number of samples (–)
DGCnetwork	Deep GRU-CNN Network	*y*_i_	True value of the i-th sample (–)
RFR	Random Forest Regression	*y*_i_ *^∗^*	Predicted value of the i-th sample (–)
GA-RF	Genetic Algorithm-Optimized Random Forest	*x*	Original data (K)
MSE	Mean Squared Error	*x’*	Result after normalizing *x* (–)
MAE	Mean Absolute Error	*Min*	Minimum training temperature at the corresponding location (K)
*R* ^2^	Coefficient of Determination	*Max*	Maximum training temperature at the corresponding location (K)
Variables		*k*	Size of the convolution kernel (–)
*u*	Solid-phase velocity vector (m/s)	*X* *_TCN_*	TCN input (–)
*ρ*	Density (kg·m^−3^)	*X_CNN_*	CNN input (–)
*ρ*_s_	Density of raw petroleum coke (kg·m^−3^)	$\sqrt{d_{k}}$	Scaling factor to stabilize the dot-product calculation (–)
*m*	Total number of volatile components (–)	*Q, K, V*	Query, Key, and Value matrices (–)
*α*	Solid-phase filling rate (–)	$QW_{i}^{Q},KW_{i}^{K},VW_{i}^{V}$	Linear projection weight matrices for the i-th attention head (–)
*ξ*	pyrolysis conversion degree (–)	*head*_i_	Output feature map of the i-th attention head (–)
*ε*_s_	Void fraction of packed petroleum coke (–)	*W*	Final linear projection weight matrix for feature concatenation (–)
*R*_i_	Mass source term of volatile release during pyrolysis (kg·m^−3^·s^−1^)		
